# NaAlg-g-AA Hydrogels: Candidates in Sustainable Agriculture Applications

**DOI:** 10.3390/gels9040316

**Published:** 2023-04-07

**Authors:** Elena Manaila, Maria Demeter, Ion Cosmin Calina, Gabriela Craciun

**Affiliations:** Electron Accelerators Laboratory, National Institute for Laser, Plasma and Radiation Physics, 409 Atomistilor St., 077125 Magurele, Romania

**Keywords:** hydrogel, electron beam, swelling, cross-linking

## Abstract

Nowadays, the degradation of agricultural soil due to various factors should be a major concern for everyone. In this study, a new sodium alginate-g-acrylic acid-based hydrogel was developed simultaneously by cross-linking and grafting with accelerated electrons to be used as soil remediation. The effect of irradiation dose and NaAlg contents on the gel fraction, network and structural parameters, sol–gel analysis, swelling power, and swelling kinetics of NaAlg-g-AA hydrogels have been investigated. It was demonstrated that NaAlg hydrogels show significative swelling power that is greatly dependent on their composition and irradiation dose; they keep the structure and are not degraded in different pH conditions and different water sources. Diffusion data revealed a non-Fickian transport mechanism (0.61–0.99) also specific to cross-linked hydrogels. The prepared hydrogels were proved as excellent candidates in sustainable agriculture applications.

## 1. Introduction

The lack of water resources, rainfall, and soil desertification through excessive exploitation (massive deforestation, overgrazing), as well as the excessive use of chemical fertilizers, are key factors that have led to the degradation of agricultural land [1]. The sustainability of agriculture is seriously hindered, mainly, by the water deficit due to the reduced amount of precipitation with the irregular spatial and temporal distribution. It is well known that the connection between the lack of water and the occurrence of water stress in plants and the consequences arising from this is the impairment of plants’ general growth and crop productivity [2,3,4]. Absorbent polymeric materials of hydrogel type represent a viable solution for reducing the water stress of crops in drought conditions, making water use more efficient by reducing losses but also for restoring soil quality [1,5]. Hydrogels are cross-linked three-dimensional polymer networks that contain hydrophilic groups and have a water absorption capacity of up to a thousand times their weight [6,7,8]. The use of these materials in agriculture has attracted attention in recent years, both due to their potential to reduce water stress and maintain soil moisture in periods of drought, and their easy use and biodegradability [6,9,10]. Thus, due to the special properties of water diffusion, hydrogels absorb a large amount of water during rain events, which they can then gradually release into the surrounding soil and rhizosphere areas uniformly and over a longer period [2,11]. The presence of hydrophilic functional groups on polymeric chains is responsible for the absorption capacity and the cross-links between them, with the insolubility in contact with water [2,12]. The nature of the materials from which they are obtained was the one that led to the first way of classifying hydrogels into natural, synthetic, and semi-synthetic. Natural or bio-based hydrogels are obtained from natural polymers, such as polysaccharides (alginate, chitosan, dextran, etc.), synthetic hydrogels from synthetic monomers (ethylene glycol, vinyl alcohol, acrylic acid, and acrylamide), and the semi-synthetic hydrogels are, of course, the result of the combination of natural and synthetic materials [2,13,14,15]. Alginates undergo enzymatic or radiolytic degradation to produce oligosaccharide that influences plant physiological activities. Oligo-alginates, considered one oligosaccharide, were found to elicit germination, shoot elongation, and root-growth-promoting activities [16]. Another classification of hydrogels is based on the method of cross-linking. These can be cross-linked chemically (chemical reactions, polymerization, graft polymerization, network formation of water-soluble polymer, and radiation cross-linking) and physically (ionic interactions, hydrogen bonds, hydrophobic). Chemical cross-linking leads to obtaining hydrogels with permanent absorption properties, while physical cross-linking leads to obtaining hydrogels with similar but temporary properties [17,18,19]. Synthetic hydrogels are less hydrophilic and show low degrees of degradability but have better mechanical resistance than those obtained from natural sources. Natural hydrogels come from renewable, non-toxic sources, are biodegradable, and have low costs, but they have certain disadvantages: they are soft, lack mechanical resistance, and can break easily [17,20]. Additionally, they are very sensitive, can be easily absorbed into plant tissues [10,21], can be easily degraded when exposed to natural UV rays, become much more sensitive to aerobic and anaerobic soil microorganisms, and can be easily degraded into water, carbon dioxide, and nitrogen compounds, elements that are present in soils [10,22]. The biological degradation of different kinds of polymers in the soil reaches a high degree of effectiveness, especially when solubility conditions are assured [10,23]. The biodegradation of acrylate-based hydrogels in municipal compost reached a rate from 1 to 9% per year under aerobic conditions, similar to the decomposition of organic matter in forest areas [10,24]. Currently, hydrogels obtained from synthetic monomers, such as acrylamide (AAm) and acrylic acid (AA), are mostly sold on the market. In addition to the reduced degree of degradability in the soil, their degradation products represent a significant potential for biological toxicity. According to some studies, the average degradation rate of poly(acrylate) hydrogels in the soil is only 0.45% after 24 weeks, and in the presence of some species of fungi, 3.20% after 14 weeks [1,25,26]. Acrylic acid salts (based on sodium or potassium) are widely used in the production and marketing of hydrogels for agriculture. They are non-toxic, have a high swelling capacity, and show a degradation rate of up to 15% per year [2]. Semi-synthetic hydrogels obtained by grafting AAm and/or AA onto a polysaccharide chain show a degree of biodegradation (using the composting method) of up to 91.77% after only 60 days [27]. To combine the specific properties of hydrogels obtained from synthetic materials (stability, good mechanical properties) with those of natural ones (low toxicity, high degradability), the production of semi-synthetic hydrogels by grafting, cross-linking, or simply blending has gained momentum in recent years, and almost all known methods of obtaining them have been tried [1,20,28,29].

The purpose of this study is to present the results obtained in cross-linking and grafting experiments by irradiation with a 6 MeV electron beam carried out to obtain semi-synthetic hydrogels based on sodium alginate (NaAlg) and acrylic acid (AA). Among all used techniques for hydrogel development, the technique with ionizing or non-ionizing radiation (gamma, electron beam, UV, and microwave) presents significant advantages (ease, quick preparation, and low production cost) compared to other chemical cross-linking methods [20,27]. The NaAlg, a linear anionic polysaccharide extracted from seaweed, was chosen as a natural base material for hydrogels due to its versatility of functionality, price, non-toxicity, and biodegradability potential [29,30]. A type of hydrogel based on sodium alginate grafted with acrylic acid, NaAlg-g-AA, was thus obtained by electron beam (e-beam) irradiation. Its physical, chemical, and structural properties were investigated through specific analyses, consisting of swelling, diffusion, network studies, and Fourier Transform Infrared Spectroscopy (FTIR). The swelling capacity was tested separately on three types of water (distilled water, tap water, and rainwater), the last two being the ones with which hydrogels used in agriculture come into contact in real life.

## 2. Results and Discussion

### 2.1. Gel Fraction and Network Parameters

The hydrogel structure and the cross-linking degree are usually dependent on the irradiation dose [31]. If natural polymers are added to the monomeric solutions, unstable structures and insufficient cross-linking degrees can be obtained by e-beam irradiation [32].

The influence of (NaAlg) content and irradiation dose on gel fraction and network parameters were investigated, and the results are presented in Table 1. All measurements were carried out in triplicate for each sample, and all values were expressed as mean values and standard deviations of three independent samples.

As shown in Table 1, the gel fraction of all hydrogels was over 85%, irrespective of NaAlg concentration and irradiation dose. However, the increase in gel fraction up to almost 95% with the irradiation dose increasing and its slight decrease with the NaAlg concentration, regardless of the irradiation dose, are obvious. NaAlg, a salt form of alginic acid, is a natural polysaccharide which degrades by the scissions of main chains when it is exposed to irradiation [33,34]. When a mixture containing NaAlg and AA is subjected to irradiation, an interpenetrating polymer network is formed, in which, at the same time, poly (AA) is chemically cross-linked. Consequently, AA is grafted on the alginate chain, and alginate is cross-linked [33,35]. However, irradiation can induce the degradation of natural polymers at doses over 20 kGy. Particularly in this case, the use of potassium persulphate (PP) as the initiator may lead to the acceleration of the degradation process of NaAlg with the increase in the irradiation dose [36]. As seen from Table 1, for each of the four concentrations of NaAlg, the increase in the irradiation dose has led to an increase in the hydrogel cross-link density. However, at the same irradiation dose, it can be observed that for 0.5 to 1.5% NaAlg, the cross-link density increased, while for 2% NaAlg, it decreased. The decrease in cross-linking degree with NaAlg concentration increase can be due to the degradation of the biopolymer and the splitting of the main chain [34,34]. Another important parameter in the characterization of hydrogel is the mesh size. The calculated mesh sizes were between 197 and 117 nm. As the cross-linking ratio increases, the mesh size decreases. Lower ξ values indicate a shorter distance between cross-linking points [37].

### 2.2. Sol–Gel Analysis

**Figure 1:**
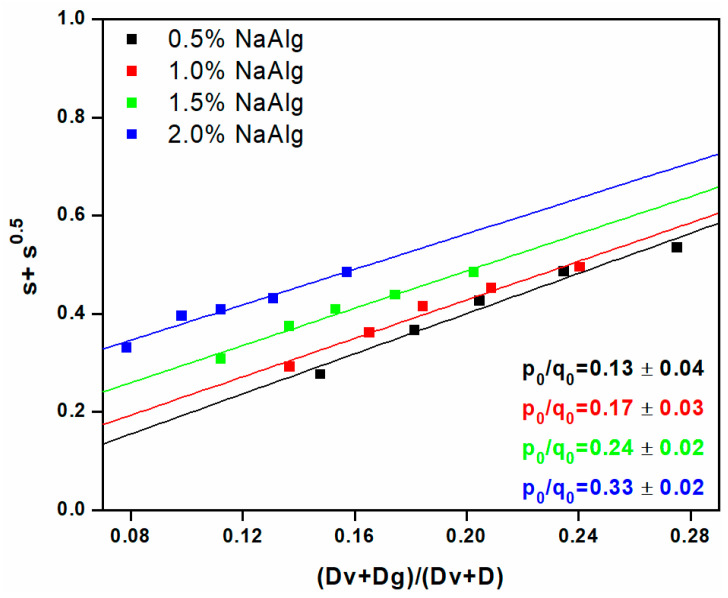
Sol–gel analysis of hydrogels according to the Charlesby–Rosiak equation.

The radiation cross-linking processes of NaAlg and AA can be highlighted using the sol–gel analysis. This is an easy and available tool to estimate the radiation cross-linking yield (G_X_) and scission (G_S_), as well as the gelation dose (Dg). Usually, the radiation cross-linking process is followed by concomitant cross-linking and degradation reactions because of the absorbed dose, polymer concentrations, and their nature. The parameters, Dg, Gx, and Gs, can be accurately estimated using a modified equation of Charlesby–Pinner, namely the Charlesby–Rosiak (Equation (10)) [38,39]. The corresponding gelation dose (Dg) and radiation degradation vs. cross-linking ratio (p_0_/q_0_/ as a function of hydrogel compositions) are shown in Figure 1 and Table 2. It is well established that in the irradiated polymer solution, the gelation point is assumed to start when at least one cross-link per each polymer chain is formed. Higher doses are needed for system gelation if the number of polymeric chains is raised. In aqueous polymer systems based on AA, the gelation dose is strongly dependent on the molar fraction of AA. As a rule, the higher the concentration of AA, the higher the gelation dose [40]. The other important parameter in the sol–gel analysis, according to Charlesby–Rosiak equations, is the virtual dose (Dv). Dv is the dose required to change the molecular weight distribution of the polymer and is an indicator of deviations existing in real systems from the assumptions made by Charlesby regarding the molecular weight distribution, as well as the random formation of cross-links.

Lower content of NaAlg requires higher Dg ~1.6 kGy, while higher content of NaAlg starts the transition from sol to gel state at 1.3 kGy. Regarding the influence of polymer concentration on the degradation vs. cross-linking (p_0_/q_0_) ratio, an increasing trend is observed. The higher polymer concentrations lead to the p_0_/q_0_ values rising to 0.33, which means a moderate increase in degradation processes. For polymeric systems which formed insoluble fractions, a higher p_0_/q_0_ ratio than 2 is associated with significant degradation processes; in the opposite sense, when p_0_/q_0_ < 2, the cross-linking processes predominated, and degradation could be considered moderate [41]. Another way to evaluate the cross-linking process induced by irradiation is to evaluate and determine the radiochemical yields of cross-linking and degradation, respectively. Thus, the radiochemical yield G (the number of cross-linking points/100 eV) is determined with Equations (11) and (12). The radiochemical yield of cross-linking and scission calculated for NaAlg hydrogels are presented in Table 3.

The G_X_ increased as the NaAlg concentration was 1.5%; above this point, the G_X_ decreased by more than an order of magnitude. In general, the NaAlg hydrogels prepared according to Sol 3 and Sol 4 compositions showed increased radiochemical degradation yields compared to those obtained for Sol 1 and Sol 2 hydrogels. Therefore, higher concentrations of Na-Alg will induce more pronounced degradative processes. In the case of Sol 3 and Sol 4 hydrogels, the ratio between the cross-linking yield and the degradation yield is higher by only two orders of magnitude, while in the case of Sol 1 and Sol 2 hydrogels, this ratio is more than three times higher. Regarding the variation of the G_X_ and G_S_ parameters with the irradiation dose, we observe that between 17.5 and 20 kGy, the G_X_ shows approximately equal values; for some compositions, there is a slightly decreasing trend. An increase in the number of cross-links will produce a decrease in the average molecular weight between two cross-links and, consequently, reduce the mesh size of hydrogels, as shown in Table 1.

The γ-irradiation of some oligosaccharides was conducted by most authors from the perspective of investigating the degradation effects induced by ionizing radiation aiming to obtain low-molecular-weight fractions of NaAlg.

In the dry state as well as in an aqueous solution, the degradation yields (G_S_) of NaAlg were found as 0.197 μmol/J and 5.7 μmol/J, respectively [34]. The γ-irradiation of 1% NaAlg resulted in a yield of scission, G_S_ of 0.55 × 10^−7^ mol/J [42], and 2% NaAlg showed a G_S_ of 0.562 μmol/J [43]. Considering the above results, we appreciate the effect induced by e-beam irradiation on NaAlg hydrogels. It is mainly attributed to the cross-linking processes.

Moreover, from the swelling experiments, it can be seen that at low concentrations of alginate, there is a proportional reduction of the water absorption capacity (distilled water, tap water, and rainwater) with the increase in the irradiation dose, which demonstrates the progressive cross-linking that takes place in the structure the newly formed hydrogel. High NaAlg concentrations show the formation of a hydrogel with fewer cross-linking points, with a structural network that easily allows the diffusion of water, as shown by swelling experiments in distilled water.

### 2.3. ATR-FTIR Analysis

**Figure 2:**
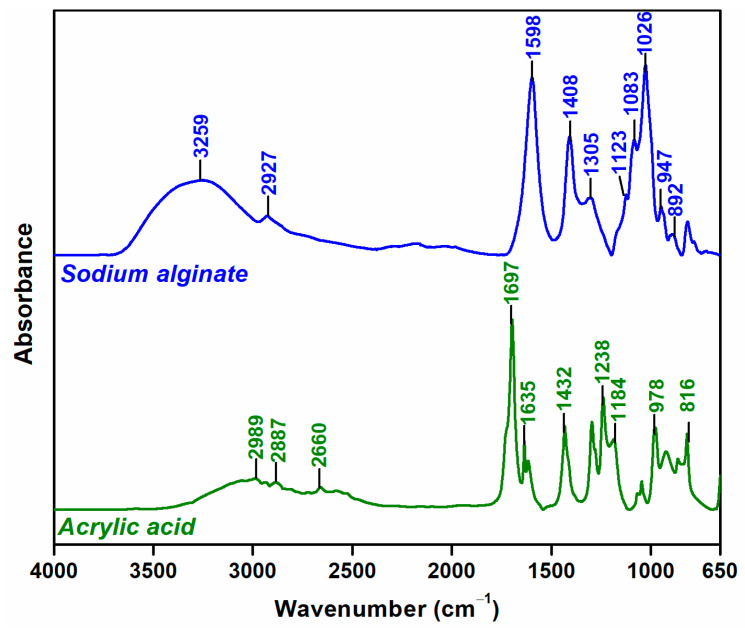
FTIR spectra of native Na-Alg and AA.

Figure 2 shows the FTIR spectra of native Na-Alg and AA. The FTIR spectra of Na-Alg showing at 3259 cm^−1^ (νO–H), 2927 cm^−1^ (νC–H), 1598 cm^−1^ (νC=O), 1408 cm^−1^ (δCOO^−^), 1305 cm^−1^ (δCH_2_), 1123 cm^−1^ (νC–O–C), and 1026 cm^−1^ (νC–O) [44].

The AA spectra indicated peaks at 2997/2887/2660 cm^−1^ (νO–H), 1697 cm^−1^ (νC=O), 1635/1615 cm^−1^ (νC=C), 1432 cm^−1^ (δCH_2_), 1238/1184 cm^−1^ (νCO, and OH of carboxylic groups), and 978/816 cm^−1^ (CH_2_ rocking mode) [45].

**Figure 3:**
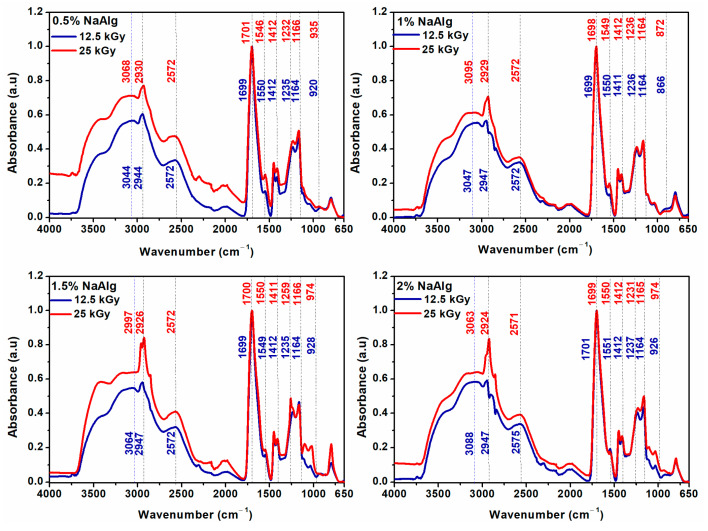
FTIR spectra of hydrogels irradiated at 12.5 kGy (D1) and 25 kGy (D5).

Chemical changes induced by the e-beam irradiation process are analyzed using ATR-FTIR. Figure 3 shows the FTIR spectra of NaAlg-g-AA hydrogels containing 0.5–2% Na-Alg and cross-linked at 12.5 kGy to 25 kGy. From the FTIR spectra of NaAlg hydrogels, it can be seen that the intensity of the bands increased at higher irradiation doses.

In the spectra of 0.5% NaAlg hydrogels, the intensity of the absorption band between the 3100–2500 cm^−1^ range was dependent on the applied irradiation dose. The O–H band was shifted from 3044 cm^−1^ to 3068 cm^−1^, and the band assigned to the C–H varied from 2944 cm^−1^ to 2930 cm^−1^ after irradiation with 25 kGy. The 1700–1600 cm^−1^ (C=O) band shows the formation of intermolecular hydrogen bonds between AA and NaAlg. The strong broad band between 1120–1000 cm^−1^ (C–O) is indicative of NaAlg. The most evident shifting was observed for peaks from 1699 cm^−1^ to 1701 cm^−1^, 1550 cm^−1^ to 1546 cm^−1^, 1235 cm^−1^ to 1232 cm^−1^, and 1164 cm^−1^ to 1666 cm^−1^. The bands from 1450/1412 cm^−1^ (COO^−^) remain unchanged.

In the case of 1% NaAlg hydrogels, the band assigned to the O-H group increased with the irradiation dose from 3047 cm^−1^ (12.5 kGy) to 3095 cm^−1^ (25 kGy). The band from 2947 cm^−1^ (C–H) was shifted to 2929 cm^−1^, and the stretching vibration of the carbonyl group (C=O) varied from 1699 cm^−1^ to 1698 cm^−1^. The other bands in the 1250–1000 cm^−1^ range remained identical regardless of the irradiation dose.

The intensity of the bands assigned to the O–H and C–H groups increased significantly for the 1.5% NaAlg hydrogel irradiated at 25 kGy. The decrease in the wavenumber from 3064/2947/1412 cm^−1^ to 2997/2926/1411 cm^−1^ is due to the cross-linking of the hydrogel at the dose of 25 kGy.

For the 2% NaAlg hydrogels, the intensity of the band at 2947 cm^−1^ increases significantly, and the band at 3088 cm^−1^ (12.5 kGy) shifts to a lower wavenumber at 3063 cm^−1^ (25 kGy).

FTIR results suggested a radiation-induced cross-linking reaction between AA and NaAlg. These results are supported by the experimental data obtained from the characterization of the network structure (Table 1), where it was shown that increasing the irradiation dose leads to an increase in the cross-linking density.

### 2.4. Swelling Kinetics and Swelling Power

To be used in agriculture, hydrogels must meet certain characteristics, including high water absorption capacity, low soluble content (i.e., high gel fraction), durability and stability during swelling (high mechanical properties), and biodegradability and reusability in several swelling–desorption cycles [5]. The hydrogels obtained by grafting AA on the NaAlg chain were obtained by cross-linking and chemical grafting through covalent bonds. They are permanent hydrogels, and they show mechanical stability, which allows them to reduce the water stress of plants over a longer time [8]. Additionally, being semi-synthetic hydrogels, they have the potential of a high degree of biodegradation, which can reach up to 90% in 60 days (through the composting method) [27].

Considering the purpose for which these hydrogels were developed, namely, to maintain soil moisture in the rooting area of crops and reduce the water stress of plants, we studied their swelling degree in three types of water: distilled water, tap water, and rainwater. Distilled water was chosen because it is usually used for laboratory analyses. We chose to carry out the same experiments in tap water and rainwater because the latter represents the most used source of water supply. All measurements were carried out in triplicate for each sample, and all values were expressed as mean values and standard deviations of three independent samples.

**Figure 4:**
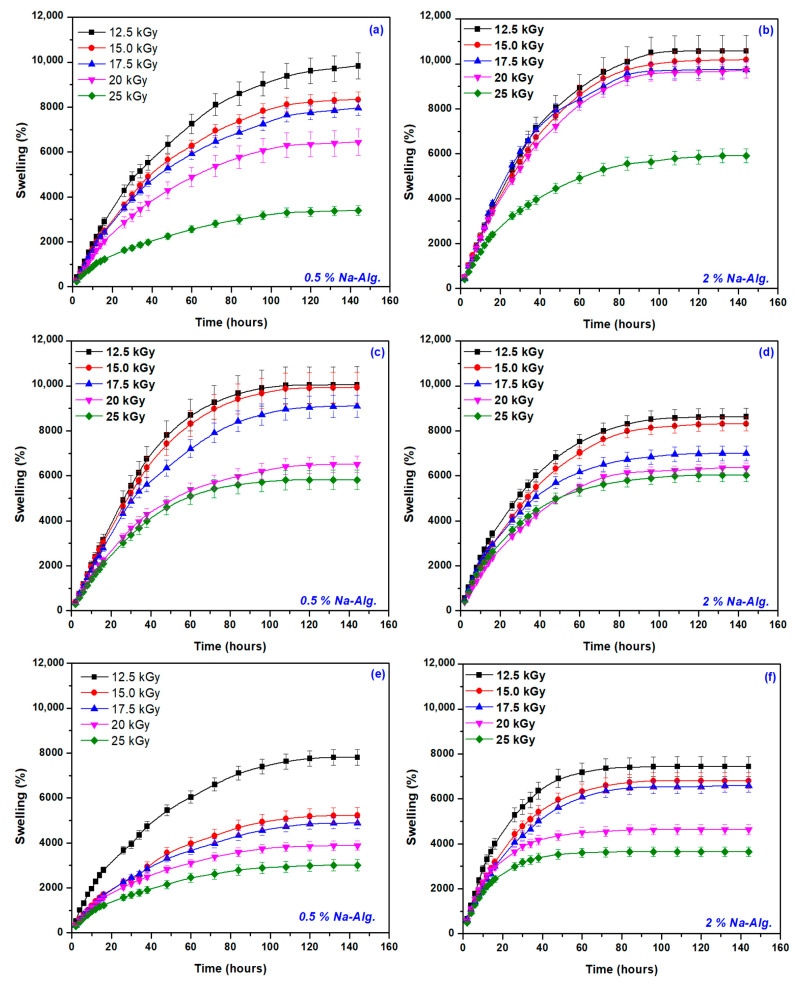
Swelling degree of hydrogels with 0.5% and 2% NaAlg in distilled water (**a**,**b**), tap water (**c**,**d**), and rainwater (**e**,**f**).

Figure 4 shows the influence of the irradiation dose on the swelling kinetics in the three types of water discussed for the mixtures with 0.5% and 2% NaAlg, respectively, and Table 4 shows the values of S_max_ for all tested hydrogels.

Comparing the 3 types of water used in the swelling experiments, we notice that for the hydrogels containing 0.5% NaAlg, the order of swelling decrease was as follows: tap water > distilled water > rainwater. For the other 3 concentrations (1, 1.5, and 2% NaAlg), the order was different: distilled water > tap water > rainwater.

As expected, increasing the cross-linking degree led to a decrease in the mesh size and a S_max_ decrease. At the lowest irradiation dose of 12.5 kGy, the highest values of S_max_ were obtained, the lowest values of S_max_ being obtained for the hydrogels obtained at the dose of 25 kGy.

The swelling degree depends on the chemical composition of the waters used in the experiments. Thus, distilled water is a type of purified water, free of salts, minerals, and other organic materials. The pH of distilled water varies from 5.8 to 7. Pure distilled water in a sealed system has a pH of 7, but as soon as it is exposed to air, it begins to absorb atmospheric gases (including CO_2_) and begins to become acidic [46]. Tap water contains salts and minerals, and their concentration differs from one area to another, depending on the geological and meteorological conditions or soil content. Some of them are called macroelements (salts of calcium, magnesium, potassium, chlorides, nitrites, nitrates, etc.) and are found in relatively large quantities of the order of mg/L, and others are called microelements (fluorine, iodine, zinc, etc.) and are found in small quantities of the order of μg/L [47]. Precipitation/rainwater, even if it is very important for the growth and development of plants, also represents a process in the removal and transport of various ionic compounds, pollutants, and soluble gases from the atmosphere to the earth’s surface [48,49]. The composition of rainwater varies from one place to another and from one region to another due to the difference between local and emission sources [48,50] and reflects all the local characteristics of atmospheric pollutants [48,51].

It is known that the degree of swelling of hydrogels is strongly influenced by chemical stimuli such as pH [19,52]. The 3 types of water used in the experiments have shown different pHs as follows: 6, 7.66, and 4.39 for distilled water, tap water, and rainwater, respectively. Swelling degrees of the hydrogels, evaluated in the 3 types of water, showed a clear dependence on the pH value, namely their increase with the increase in pH, for the mixtures with 0.5 and 1% NaAlg. The swelling of hydrogels is due to the presence of strongly hydrophilic groups in the structure, namely carboxylic (-COOH) and hydroxyl (-OH) groups [53]. The degree of protonation of the carboxylic groups is closely related to the pH of the swelling medium, which gives the hydrogel its pH response. When the average pH increases, most of the carboxylic groups are negatively charged, they become more extended, and this extension facilitates the diffusion of water molecules in the hydrogel network. Otherwise, at low pH, the hydroxyl groups are mostly in the protonated form and present a less polar character and, therefore, result in a polymer with a lower affinity to water, hence a lower swelling degree [54]. The swelling degree is also influenced by the presence of cations such as Na^+^, K^+^, Mg^2+^, and Ca^2+^ that are found in tap water and rainwater. The degree of swelling in rainwater is the lowest. This results from a charge-shielding effect of the additional cations that cause an anion–anion electrostatic repulsion, which, in turn, leads to a decrease in the osmotic pressure difference between the polymer network and the external solution. At a given ionic strength, Mg^2+^ and Ca^2+^ contribute with more charge than the monocovalent cations Na^+^ and K^+^ and induce a greater decrease in intermolecular repulsion and increased interaction between molecules, which in turn greatly cause hydrogel collapse. In addition, Mg^2+^ and Ca^2+^ can chelate the carboxylic group, leading to a compact network and causing further shrinking of the hydrogel [54].

The types of ions present in the system influence the formation of hydrogen bonds with different resistances, a fact that can be explained based on the hydration model of the hydrogen bonds of the polymer side chains [54,55]. According to this model, ion-specific swelling behavior is caused by the stabilization or destabilization of hydrogen bond hydration by ionic hydration. In the case of hydration to anions, the positive charge of water oxygen decreases, and the negative charge of water oxygen increases. These changes in the charges on the water hydrogen and oxygen due to hydration to ions correspond to a decrease in the ability of electron-pair acceptance (Type A) and an increase in the ability of electron-pair donation (Type B) of water, respectively. In tap water and rainwater, the presence of cations (K^+^, Na^+^, Mg^2+^, and Ca^2+^) improves the strength of the hydrogen bond in the hydrogel because more electrical charges are pushed toward the oxygen molecules of the O-H bond in the water, molecules that increase the capacity of the hydrogen atom of water to accept the electrical pairs donated by the oxygen atoms in the hydrogel, which becomes more stable (type A) [54]. At the same time, the existence of Cl^−^, NO^2−^, and NO^3−^ anions make the water molecule serve as a hydrogen bond acceptor. B-type hydrogen bonding hydration is stabilized by anion hydration due to the enhancement of water electron pair donation [54].

**Figure 5:**
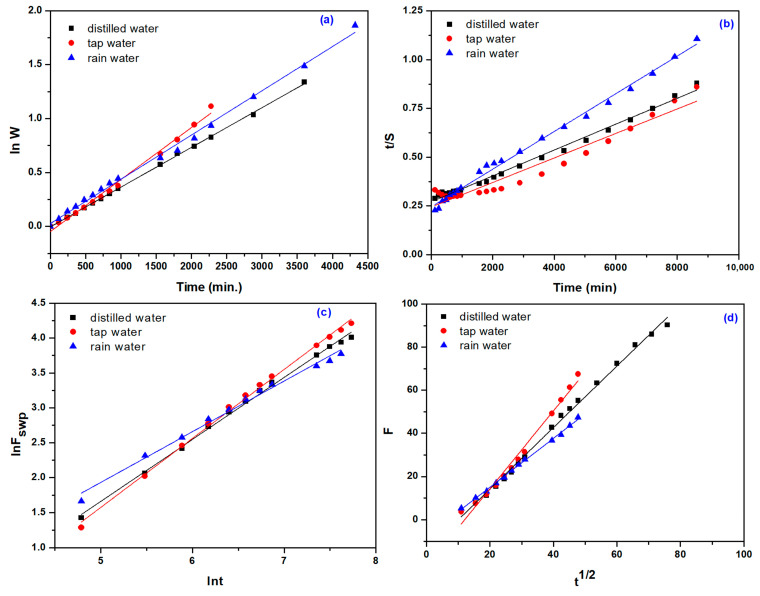
The first-order swelling kinetics (**a**); The second-order swelling kinetics (**b**); The plots of lnF versus lnt (**c**); Plots of F versus t1/2 (**d**) for 0.5% NaAlg, 12.5 kGy, and all the waters taken in the experiments.

To investigate the controlling mechanism of the swelling processes, two kinetic models were used: the first-order equation and the second-order equation based on the swelling equilibrium degree. The rate constant of first-order swelling (k_1,s_), the initial swelling rate (r_o_/g water (g gel min)^−1^), and equilibrium swelling (S_max_/g water (g gel)^−1^) were determined, and the results are presented in Figure 5 and Table 5 and Table 6. The determinations were made on the swelling dynamics averaged based on three determinations (see Figure 4).

The swelling rate constant, k_1,s_, of first order (Table 5) shows increasing values with the increase in NaAlg concentration (up to 1.5%) and decreasing values with the increase in irradiation dose up to 17.5 kGy. For tap water and rainwater, the values are higher than for distilled water, regardless of the hydrogel type. The increase in k_1,s_ for the hydrogels obtained at the irradiation doses of 20 and 25 kGy can be explained by the greater proportion of degradation reactions compared to those of grafting/cross-linking. The same explanation can be applied in the case of hydrogels containing 2% NaAlg. This result is also supported by the sol–gel analysis (Table 2). Moreover, some studies have reported that an increase in the degree of swelling is correlated with low values of k_1,s_ [56,57].

Table 6 shows the initial swelling rate (r_0_/g water (g gel min)^−1^) and equilibrium swelling (S_max_/g water (g gel)^−1^) calculated according to the second-order swelling equation.

Although it was expected that the hydrogels with a larger amount of NaAlg would swell faster due to the increased degree of hydrophilicity, this was not the case. Regarding the initial swelling rate (r_0_), it decreased with increasing NaAlg concentration and increased with increasing irradiation dose, while S_max_ presented values inversely proportional to r_0_ (increasing values with increasing NaAlg concentration and decreasing with the radiation dose). An explanation can be linked to the fact that the degree and speed of swelling of hydrogels largely depend on the ionization of the strongly hydrophilic groups in the structure, the carboxylic (-COOH) and hydroxyl (-OH) groups [58].

Because the diffusion exponents (n) are indicative of the transport mechanism, for all hydrogels, lnF vs. lnt graphs were plotted, diffusion exponents (n) and diffusion constant (k) were calculated from the slopes and intercepts, and the results are listed in Table 7.

According to the literature [54,59], n values can be as follows: (i) n < 0.45–0.5 for Fickian diffusion or Case I, where the rate of solvent diffusion is much lower compared to the macromolecular chain relaxation of the polymer [32]; (ii) 0.45–0.5 < n < 1.0 for non-Fickian diffusion or anomalous (in which the diffusion of the solvent into the hydrogel structure is fast compared to the relaxation rate of the macromolecular chain of the polymer [32]; (iii) n = 1.0 for zero order or Case II, where the diffusion process is much faster than the relaxation process [32]; and (iv) n > 1.0 for super Case II type of penetrating mechanism. The anomalous case is a summation of Fickian diffusion (Case I) and non-Fickian diffusion (Case II) [54,60].

As seen from Table 7, the values of the diffusion exponent (n) vary between 0.61 and 0.99, which corresponds to a diffusion mechanism of non-Fickian type, in which the diffusion and relaxation rates are comparable. As seen clearly, the n values of hydrogels immersed in rainwater (pH 4.39) were lower compared to those immersed in distilled and tap water (pH of 6.0 and 7.66, respectively). With the pH increasing, it is observed that the absorption profile becomes more dependent on polymer diffusion, an effect that may be attributed to the protonation of the carboxylic group at low pH, which causes the formation of hydrogen bonds with the hydroxyl group and can pull the polymer chains together to form a tight network, thus reducing the swelling degree. The increase in pH leads to the formation of carboxylate ions that cause repulsion between molecular chains and this leads to an increase in the swelling degree [54].

As seen from Table 8, the values of the diffusion coefficient (D) vary from 0.13 × 10^−3^ to 2.04 × 10^−3^ cm^2^/s, depending on the irradiation dose and the composition of the hydrogel.

The diffusion coefficient is dependent on the concentration of NaAlg, the irradiation dose, and the pH of the immersion solution. An increase in the diffusion coefficient is observed with increasing NaAlg concentration and a decrease with increasing irradiation dose. The result is also correlated with the one obtained for mesh size, as shown in Table 1. The relatively large size of the hydrogel meshes contributes to the faster penetration of water into the porous structure of the hydrogel [61]. On the other hand, the faster transport of water in the hydrogel network can also be caused by the osmotic pressure of the gel that must be permanently overcome by water during the swelling process. Thus, osmotic pressure may be responsible for the identified non-Fickian water transport mechanism [62,63]. The lowest values of the diffusion coefficient were obtained in the case of rainwater, which also has the lowest pH.

**Figure 6:**
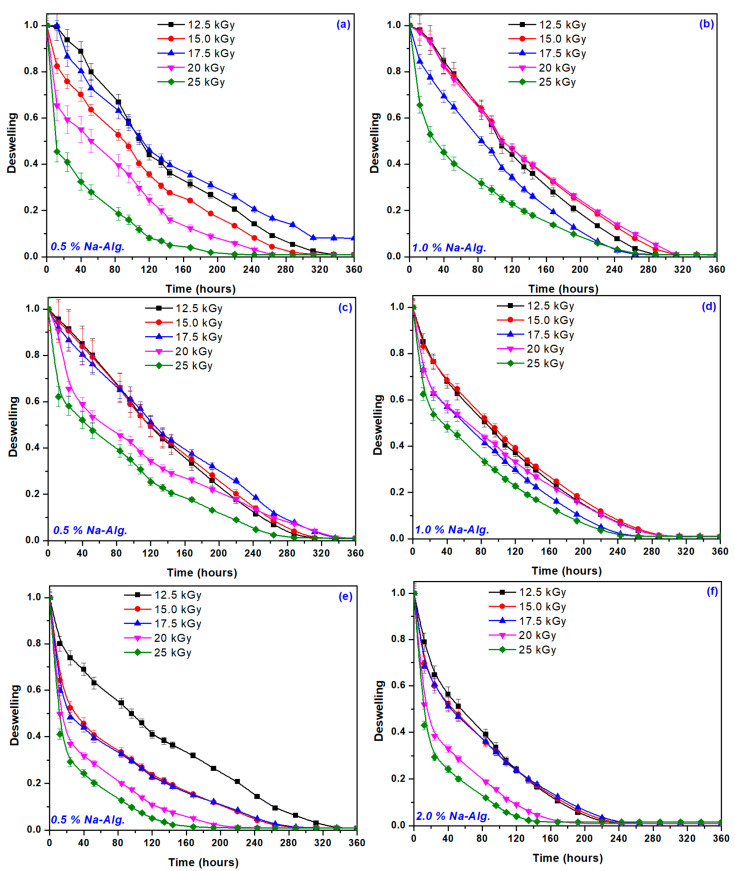
Deswelling degree of hydrogels with 0.5% and 2% NaAlg in distilled water (**a**,**b**), tap water (**c**,**d**), and rainwater (**e**,**f**).

Figure 6 shows the deswelling curves of hydrogels with 0.5 and 2.0% NaAlg content for all irradiation doses.

Based on the results presented in Figure 6, a clear dependence of the degree of deswelling on the irradiation dose and the NaAlg content can be observed, also recorded in the studies of the gel fraction, cross-linking density, and polymer network. The deswelling degree was up to 50% in 120 h for distilled and tap water and 80 h for rainwater. For hydrogels obtained at the irradiation dose of 12.5, 15, and 17 kGy, the smaller the gel fraction, the larger the pore sizes, and the deswelling process occurred more slowly.

## 3. Conclusions

In this work, NaAlg-g-AA hydrogels of different compositions were successfully prepared using e-beam irradiation. The results of this study demonstrated that the irradiation dose and the NaAlg concentration have a significant effect on the physicochemical properties of NaAlg-g-AA hydrogels.

The cross-linking, swelling power, and swelling kinetics were mainly influenced by the irradiation dose and the water pH. The swelling decrease demonstrates the progressive cross-linking that takes place in the structure of the newly formed hydrogel. Higher pH values produced a swelling increase in hydrogels below and at 1% NaAlg content.

The gel fraction increased with increasing the irradiation dose up to 94.9%. However, at low doses of irradiation, the increase in NaAlg concentration led to an increase in gel fraction, while at high irradiation doses, the increase in NaAlg concentration led to a slight decrease.

The mesh size values ranged from 117 nm (at 15 kGy) to 197 nm (at 12.5 kGy), indicating a shorter distance between cross-linking points, which allows more than 10,000% water absorption in the hydrogel network, suitable for the targeted application. The porosity of the formed network was slightly influenced by both NaAlg concentration and irradiation dose. The evaluation of the cross-linking process showed that lower content of NaAlg requires a higher gelation dose (~1.6 kGy), while higher content of NaAlg starts the transition from sol to gel state at 1.3 kGy.

The swelling exponents were found in the range of 0.61–0.99, thus suggesting a non-Fickian diffusion mechanism that is characteristic of cross-linked hydrogels.

The FTIR analysis highlighted the formation of a miscible mixture between NaAlg and AA by increasing the intensity of the bands and their shifting to lower wavelengths due to the cross-linking process.

The results of the diffusion experiments carried out on three different types of water showed that in rainwater, the swelling was higher than in distilled and tap water due to the presence of cations, such as K^+^, Na^+^, Mg^2+^, and Ca^2+^, which improve the strength of the hydrogen bond in the hydrogels.

## 4. Materials and Methods

### 4.1. Materials

Sodium alginate (SA, M_w_ = 216.121 g/mol, density = 1.601 g/cm^3^), acrylic acid (AA, M_w_ = 71.08 g/mol, density = 1.13 g/cm^3^) and potassium persulfate (PP, M_w_ = 270.322 g/mol, density = 2.477 g/cm^3^) have been used for samples preparation. These were purchased from Merck KGaA, Darmstadt, Germany, and used without further purification.

### 4.2. Experimental Installation and Samples Preparation

Irradiation using an electron beam of 5.5 MeV was used as grafting method for obtaining hydrogels. The linear accelerator that has been used is called ALID 7, its owner being the Electron Accelerators Laboratory from the National Institute for Lasers, Plasma and Radiation Physics, Bucharest, Romania.

In any irradiation process, the rigorous control of irradiation dose and dose rate is mandatory, and for this purpose, the primary standard graphite calorimeter has been used. Four types of aqueous solutions were prepared as in Table 9. These have been placed in medical syringes with a diameter of 1.5 mm and irradiated with 12.5 kGy (D_1_), 15 kGy (D_2_), 17.5 kGy (D_3_), 20 kGy (D_4_), and 25 kGy (D_5_) in atmospheric conditions and at room temperature of 25 °C, the process dose rate being of 2.4 kGy/min.

### 4.3. Sodium Alginate-g-Acrylic Acid Hydrogels Characterization

For being analyzed, the hydrogels obtained as above were left to dry in air for 24 h and were then cut into round pieces with thickness of 3–4 mm that were brought to constant mass by drying in the air for 3 days, followed by climbing in a laboratory oven for 24 h at 50 °C.

#### 4.3.1. Gel Fraction and Swelling

For soluble fraction removal, dried samples of about 0.05 g were placed in distilled water at room temperature of 25 °C for 24 h, then dried in air and finally dried in a laboratory oven for 24 h at 50 °C to constant weight. The gel fraction was calculated using the following equation [64]:

(1) $$Gel-fraction(\%)=\frac{m_{f}}{m_{i}}\times 100,$$

where *m_i_* is the initial weight of the dried sample, and *m_f_* is the weight of the dried sample after extraction from the water.

Swelling experiments have been conducted in distilled water at room temperature of 25 °C. The mass increases until the equilibrium reaches being evaluated by regular weighing. Swollen hydrogels at equilibrium are then left in the open air in order to study their deswelling, and the mass changes of hydrogels or water retention were calculated by measuring the mass of each sample at regular times. The swelling *S*(%) was calculated from relation (2) [58,65] and the deswelling from relation (3) [66]:

(2) $$S(\%)=\frac{m_{t}-m_{i}}{m_{i}}\times 100,$$

(3) $$Deswelling=\frac{m_{t}}{m_{i}}\times 100,$$

where *m_t_* is the mass of the swollen gel at time *t*, and *m_i_* is the initial mass of the dried gel.

#### 4.3.2. Network Parameters

The polymer cross-link density, *q*, has been calculated as the ratio between the molecular weight of the polymer repeating units (*M*_0_) and the average molar mass between cross-links (*M_c_*) [67,68,69]:

(4) $$q=\frac{M_{0}}{M_{c}}.$$

*M*_0_ was calculated using the following equation [68,69,70]:

(5) $$M_{0}=\frac{\left(m_{SA}\times M_{SA}\right)+\left(m_{AA}\times M_{AA}\right)}{m_{SA}+m_{AA}},$$

where *m_SA_* and *m_AA_* are the masses of *SA* and *AA* expressed in grams, and *M_SA_* and *M_AA_* are the molar masses of *SA* and *AA* expressed in g mol^−1^.

*M_c_* has been thus calculated using the Flory—Rehner theory for perfect networks [58]:

(6) $$M_{c}=-V_{1}d_{P}\frac{\nu_{S}^{1/3}-\nu_{S}/2}{\ln(1-\nu_{S})+\nu_{S}+\chi \nu_{S}^{2}},$$

where *V*_1_ is the molar volume of water (18 cm^3^ mol^−1^), *d_P_* is the polymer density, *υ_S_* is the volume fraction of the polymer in the swollen gel (cm^3^), and *χ* is the Flory–Huggins interaction parameter between the solvent and polymer. The sample densities, determined using an electronic balance equipped with kits for density determination (AS 220, 0.1 mg resolution, producer Radwag, Warsaw, Poland), were between 1.045 and 1.285.

The value of *χ* was calculated using the following equation:

(7) $$\chi =0.431-0.311\nu_{S}-0.036\nu_{S}^{2}.$$

Based on the values of *M_c_* calculated as above and knowing the volume fraction of the polymer in the swollen gel (*υ_S_*), the length of the C–C bond along the polymer backbone (*l* = 0.154 nm), the Flory characteristic ratio of the polymer (*C_n_*), and the molecular mass of repeated unit (*M_r_*), the mesh size (*ξ*) was determined using the following equation [71]:

(8) $$\xi =\upsilon_{s}^{-1/3}l\sqrt{\frac{2C_{n}M_{c}}{M_{r}}},$$

*C_n_* was taken as being the weighted average of *C_n_* of poly(AA) and SA chains, according to their molar ratio in the hydrogel. The *C_n_* values of poly(AA) and SA were 6.7 and 21.1, respectively [72,73].

The porosity of the hydrogels, *P*(%), was determined as a function of the volume ratio of water at equilibrium (*V_d_*) using the following relation [58]:

(9) $$P(\%)=\frac{V_{d}}{1-V_{d}}\times 100.$$

#### 4.3.3. Sol–Gel Analysis

The free available software, Gelsol95, was used for the determination of gelation dose and degradation vs. cross-linking ratios of the hydrogels. The calculation is based on the Charlesby–Rosiak equation [39]:

(10) $$s+\sqrt{s}=\frac{p_{0}}{q_{0}}+\left(2-\frac{p_{0}}{q_{0}}\right)\left(\frac{D_{V}+D_{g}}{D_{V}+D_{}}\right),$$

where *s* is the sol fraction, *p*_0_ is the degradation density, *q*_0_ is the cross-linking density, *D* (kGy) is the irradiation dose, *D_g_* (kGy) is the gelation dose, and *D_V_* (kGy) is the virtual dose necessary to transform the real sample into a sample with the molecular weight distribution of M_w_/M_n_ = 2.

The radiation yields of cross-linking (*G_X_*) and degradation (*G_S_*) were calculated using the following equations [39]:

(11) $$G_{X}=\frac{4.9\cdot 10^{2}\cdot c}{M_{C}\cdot D\cdot \rho },$$

(12) $$G_{S}=G_{X}\cdot 2\frac{p_{0}}{q_{0}},$$

where *G_X_* is expressed as the number of moles of cross-linking bonds per Joule, *G_S_* is the radiation yield of chain scission (mol/J), *M_c_* (g/mol) is the average molecular weight between two successive cross-links, *c* (g/L) is the polymer concentration in the irradiated solution, *D* (J/kg) is the absorbed dose, and ρ (kg/m^3^) is the polymer density.

#### 4.3.4. ATR-FTIR Analysis

The presence of functional groups from sodium alginate and acrylic acid in the hydrogels was investigated by FTIR measurements (30 scans/sample) using the Spectrum 100 instrument (Perkin Elmer, Waltham, MA, USA) in ATR mode at a resolution of 4 cm^−1^ and in the range of 4000–650 cm^−1^. All spectra were analyzed using Spectrum v. 6.3.2 software.

#### 4.3.5. Swelling Kinetics and Swelling Power

The water diffusion mechanisms were investigated based on the results obtained in swelling experiments and first- and second-order swelling kinetics, where the first-order swelling constant (*k*_1,*S*_) and degree of swelling at equilibrium (*S*_max._) were determined [58]:

(13) $$\frac{dS}{dt}=k_{1,S}\left(S_{\max.}-S\right).$$

By applying the initial condition, *S* = 0 at *t* = 0 and *S* = *S* at *t* = *t*, the equation becomes:

(14) $$\ln W=k_{1,S}t,$$

(15) $$W=\frac{S_{\max.}}{S_{\max.}-S}.$$

The second-order swelling constant (*k*_2,*S*_) was determined from the following equation [58]:

(16) $$\frac{dS}{dt}=k_{2,S}{\left(S_{\max.}-S\right)}^{2}.$$

By applying the initial condition, *S* = 0 at *t* = 0 and *S* = *S* at *t* = *t*, the equation becomes:

(17) $$\frac{t}{S}=A+Bt,$$

where *A* is the reciprocal of the initial swelling rate, and *B* is the inverse of the swelling degree at equilibrium:

(18) $$A=r_{0}=\frac{1}{k_{2,S}\times S_{\max.}{}^{2}},$$

(19) $$B=\frac{1}{S_{\max.}},$$

The first- and second-order equations have been used to calculate the swelling kinetic parameters, theoretical degree swelling at equilibrium (*S*_max._), and the initial swelling rate (*r*_0_).

Depending on the application for which they are produced, hydrogels must present certain specific characteristics of water diffusion in the polymer network, their evaluation being conducted through swelling experiments. The manner of water diffusion in hydrogels can be evaluated by applying the following equations to 60% of swelling curves [74,75]:

(20) $$F_{swp}=\frac{M_{t}-M_{0}}{M_{0}}=kt^{n},$$

(21) $$\ln F_{swp}=n\ln t+\ln k,$$

where *M_t_* and *M*_0_ are the masses of the swollen and dry samples at time *t*, *k* is the swelling constant, and *n* is the swelling exponent.

The diffusion coefficient (*D*) was calculated by the short-time approximation method that is valid only for the first 60% of the swelling, using the following equation [58]. The diffusion coefficients have been calculated using the following relation:

(22) $$F=4{\left[\frac{D}{\pi \times r^{2}}\right]}^{1/2}t^{1/2},$$

where *D* is in cm^2^s^−1^, *t* in *s*, and *r* is the radius of cylindrical polymer sample (cm).

## Figures and Tables

**Table 1:** Gel fraction and network parameter values.

Dose	NaAlg	Gel Fraction	Mc	q × 10^4^	ξ	P
(kGy)	(%)	(%)	(g/mol)		(nm)	(%)
12.5	0.5	85.09 ± 0.47	1,280,778 ± 9185	0.61 ± 0.01	197 ± 5.20	99.03 ± 0.01
	1.0	86.81 ± 2.41	1,104,867 ± 209,444	0.73 ± 0.21	180 ± 10.58	98.93 ± 0.16
	1.5	87.23 ± 0.71	760,629 ± 87,288	1.10 ± 0.16	141 ± 11.55	98.74 ± 0.10
	2.0	87.26 ± 1.99	978,774 ± 366,273	0.89 ± 0.19	169 ± 13.61	98.93 ± 0.15
15	0.5	87.16 ± 0.82	1,259,597 ± 171,879	0.62 ± 0.10	195 ± 19.00	99.02 ± 0.09
	1.0	88.57 ± 1.42	1,034,249 ± 141,578	0.78 ± 0.14	172 ± 17.01	98.89 ± 0.11
	1.5	89.12 ± 1.81	675,868 ± 251,093	1.24 ± 0.28	130 ± 10.15	98.65 ± 0.20
	2.0	89.41 ± 3.71	912,866 ± 386,402	0.95 ± 0.22	161 ± 15.53	98.89 ± 0.17
17.5	0.5	89.59 ± 0.62	942,176 ± 69,847	0.82 ± 0.07	158 ± 8.08	98.80 ± 0.06
	1.0	90.06 ± 0.53	971,382 ± 127,415	0.83 ± 0.15	163 ± 15.14	98.81 ± 0.14
	1.5	90.26 ± 1.38	597,842 ± 135,890	1.40 ± 0.23	118 ± 15.28	98.51 ± 0.15
	2.0	90.30 ± 1.81	853,962 ± 231,492	1.02 ± 0.18	153 ± 16.17	98.81 ± 0.13
20	0.5	91.87 ± 1.82	912,356 ± 110,643	0.85 ± 0.09	153 ± 12.66	98.74 ± 0.08
	1.0	92.04 ± 1.95	889,683 ± 114,085	0.91 ± 0.10	152 ± 13.00	98.71 ± 0.08
	1.5	91.55 ± 1.93	566,062 ± 301,028	1.48 ± 0.41	113 ± 8.19	98.42 ± 0.28
	2.0	90.77 ± 2.26	731,308 ± 183,880	1.19 ± 0.21	136 ± 17.93	98.66 ± 0.14
25	0.5	94.90 ± 1.47	661,076 ± 145,925	1.17 ± 0.47	122 ± 9.54	98.45 ± 0.31
	1.0	94.40 ± 2.80	637,864 ± 96,165	1.27 ± 0.27	120 ± 13.20	98.40 ± 0.18
	1.5	93.88 ± 1.89	556,987 ± 32,206	1.51 ± 0.08	110 ± 4.62	98.36 ± 0.05
	2.0	93.13 ± 1.73	598,177 ± 5168	1.45 ± 0.01	117 ± 6.35	98.46 ± 0.01

**Table 2:** Sol–gel parameters calculated according to Charlesby–Rosiak.

Composition	p_0_/q_0_	D_g_ (kGy)	D_v_ (kGy)	R^2^
Sol 1 (0.5% NaAlg)	0.13	1.60	2.50	0.96
Sol 2 (1.0% NaAlg)	0.17	0.11	3.96	0.97
Sol 3 (1.5% NaAlg)	0.24	1.10	2.00	0.97
Sol 4 (2.0% NaAlg)	0.33	1.31	1.00	0.95

**Table 3:** Radiochemical yield of cross-linking (G_X_) and scission (G_S_) calculated for hydrogels.

Dose (kGy)	Radiochemical Yields (mol/J × 10^−10^)	Sol 1 (0.5% NaAlg)	Sol 2 (1.0% NaAlg)	Sol 3 (1.5% NaAlg)	Sol 4 (2.0% NaAlg)
12.5	G_X_	4.62	5.44	6.55	4.96
	G_S_	1.28	1.92	3.20	3.27
15	G_X_	3.88	4.86	5.92	4.17
	G_S_	1.07	1.71	2.89	2.75
17.5	G_X_	4.47	4.44	5.49	4.80
	G_S_	1.24	1.56	2.68	3.17
20	G_X_	4.07	4.25	5.51	4.37
	G_S_	1.13	1.50	2.69	2.88
25	G_X_	4.43	4.69	5.28	4.76
	G_S_	1.23	1.65	2.58	3.14

**Table 4:** The hydrogel maximum water uptake, S_max_ (%) at equilibrium.

NaAlg (%)	Dose (kGy)	S_max_ (%)		
		Distilled Water (Water 1)	Tap Water (Water 2)	Rainwater (Water 3)
0.5%	12.5	9831 ± 586	10,047 ± 807	7806 ± 345
	15	8330 ± 335	9924 ± 695	5224 ± 355
	17.5	7953 ± 315	9093 ± 498	4877 ± 240
	20	6438 ± 591	6515 ± 360	3881 ± 208
	25	3396 ± 215	5821 ± 432	3011 ± 253
1.0%	12.5	10,117 ± 623	9257 ± 666	7556 ± 436
	15	9878 ± 640	7960 ± 322	5701 ± 469
	17.5	9860 ± 428	7206 ± 439	4891 ± 209
	20	6967 ± 374	6879 ± 357	3998 ± 167
	25	4411 ± 405	5904 ± 217	2942 ± 186
1.5%	12.5	9129 ± 636	7968 ± 440	5552 ± 437
	15	8165 ± 532	7534 ± 333	5286 ± 354
	17.5	7650 ± 283	7063 ± 356	4673 ± 297
	20	6612 ± 233	7054 ± 438	3926 ± 230
	25	4710 ± 342	5240 ± 249	3249 ± 213
2.0%	12.5	10,572 ± 696	8623 ± 375	7439 ± 439
	15	10,182 ± 459	8311 ± 305	6805 ± 355
	17.5	9746 ± 376	6992 ± 334	6588 ± 298
	20	9726 ± 358	6354 ± 207	4636 ± 228
	25	5911 ± 306	6030 ± 289	3645 ± 218

**Table 5:** First-order swelling rate constants k_1,S_ × 10^4^/min^−1^.

Dose (kGy)	Water Type	0.5% NaAlg		1% NaAlg		1.5% NaAlg		2% NaAlg	
		k_1,S_	R^2^	k_1,S_	R^2^	k_1,S_	R^2^	k_1,S_	R^2^
12.5	Water 1	3.68	0.999	4.42	1.000	6.39	0.999	5.14	0.996
	Water 2	4.82	0.992	4.07	0.998	4.74	0.999	5.12	0.999
	Water 3	4.10	0.996	6.25	0.998	6.95	0.995	9.84	0.983
15	Water 1	3.92	0.999	4.18	0.999	6.48	0.998	5.08	0.991
	Water 2	4.43	0.995	3.65	0.998	4.67	0.997	4.63	0.999
	Water 3	3.91	0.998	7.01	0.987	7.88	0.988	7.71	0.992
17.5	Water 1	3.80	1.000	3.82	0.998	6.42	0.997	5.77	0.997
	Water 2	4.32	0.999	4.07	0.999	4.76	0.994	5.51	0.998
	Water 3	3.80	0.998	6.29	0.996	5.58	0.997	7.28	0.984
20	Water 1	3.85	0.999	4.53	0.998	6.26	0.983	4.99	0.992
	Water 2	4.68	0.999	3.88	0.999	5.52	0.997	4.74	0.999
	Water 3	4.44	0.995	5.13	0.996	5.89	0.996	9.24	0.994
25	Water 1	3.66	0.994	5.21	0.996	6.01	0.998	4.84	0.999
	Water 2	5.00	0.998	4.89	0.998	4.27	0.998	5.77	0.999
	Water 3	4.38	0.992	6.29	0.995	10.30	0.998	12.00	0.998

**Table 6:** Second-order swelling rate constants, values of initial swelling rate (r_0_ × 10^2^/g water (g gel min)^−1^), and equilibrium swelling (S_max_/g water (g gel)^−1^).

Dose (kGy)	Water Type	0.5% NaAlg			1.0% NaAlg			1.5% NaAlg			2.0% NaAlg		
		r_0_	S_max_	R^2^	r_0_	S_max_	R^2^	r_0_	S_max_	R^2^	r_0_	S_max_	R^2^
12.5	Water 1	27.08	150.89	0.992	22.03	146.22	0.985	14.72	159.93	0.989	20.06	153.55	0.978
	Water 2	24.40	159.24	0.939	27.07	143.69	0.979	24.30	110.79	0.987	19.23	113.25	0.992
	Water 3	24.49	103.55	0.994	15.71	90.79	0.997	16.93	64.06	0.998	13.00	87.51	0.992
15	Water 1	31.37	129.18	0.985	22.32	140.54	0.998	15.65	100.44	0.990	20.41	145.61	0.983
	Water 2	25.19	156.96	0.958	29.77	116.49	0.992	25.21	102.75	0.993	23.66	115.70	0.988
	Water 3	40.29	71.54	0.992	19.89	68.44	0.996	18.31	61.62	0.997	17.75	83.23	0.992
17.5	Water 1	32.05	118.82	0.991	23.39	141.20	0.996	16.77	108.24	0.990	19.72	137.10	0.967
	Water 2	27.28	140.30	0.975	32.04	106.60	0.988	27.76	98.29	0.989	20.82	88.24	0.995
	Water 3	41.94	65.71	0.993	23.60	58.37	0.998	26.44	55.93	0.998	18.70	80.22	0.994
20	Water 1	37.80	95.39	0.992	26.93	93.33	0.996	18.68	113.13	0.992	21.00	137.29	0.985
	Water 2	31.78	91.40	0.990	34.43	100.95	0.994	22.32	91.97	0.989	28.52	85.59	0.990
	Water 3	40.68	48.71	0.997	28.98	47.11	0.999	32.36	47.37	0.998	13.38	51.21	0.998
25	Water 1	54.75	43.75	0.993	31.57	54.28	0.998	25.39	58.26	0.998	27.71	75.81	0.997
	Water 2	33.63	81.78	0.981	32.12	81.23	0.988	35.89	70.78	0.994	22.93	75.09	0.995
	Water 3	49.27	36.96	0.994	31.90	33.79	0.999	17.01	35.60	0.997	15.21	39.95	0.997

**Table 7:** The values of swelling exponents, *n*, and swelling constants, *k* × 10^2^.

Dose (kGy)	Water Type	0.5% NaAlg			1.0% NaAlg			1.5% NaAlg			2.0% NaAlg		
		k	n	R^2^	k	n	R^2^	k	n	R^2^	k	n	R^2^
12.5	Water 1	6.17	0.89	0.997	6.36	0.91	0.991	11.35	0.85	0.973	7.39	0.90	0.998
	Water 2	3.43	0.99	0.997	5.46	0.90	0.998	8.15	0.85	0.995	13.34	0.80	0.994
	Water 3	18.24	0.73	0.990	18.37	0.76	0.985	22.23	0.71	0.977	17.97	0.78	0.982
15	Water 1	4.35	0.92	0.994	9.75	0.84	0.993	11.50	0.84	0.977	8.75	0.87	0.996
	Water 2	4.28	0.95	0.998	10.27	0.79	0.999	8.71	0.83	0.994	9.03	0.84	0.996
	Water 3	11.77	0.72	0.999	21.42	0.70	0.987	19.85	0.71	0.983	15.49	0.77	0.996
17.5	Water 1	4.64	0.90	0.991	8.33	0.86	0.993	10.31	0.84	0.974	5.38	0.94	0.993
	Water 2	4.43	0.93	0.996	6.11	0.86	0.997	7.34	0.84	0.997	13.28	0.78	0.986
	Water 3	11.06	0.72	0.997	18.92	0.69	0.987	17.39	0.70	0.996	22.17	0.71	0.997
20	Water 1	4.81	0.83	0.991	8.97	0.82	0.999	10.13	0.83	0.983	9.29	0.85	0.997
	Water 2	5.48	0.87	0.996	6.52	0.84	0.997	9.83	0.82	0.991	8.47	0.81	0.994
	Water 3	12.58	0.70	0.990	21.95	0.64	0.989	15.76	0.68	0.996	29.91	0.66	0.964
25	Water 1	10.36	0.68	0.987	12.02	0.73	0.979	12.98	0.74	0.977	11.22	0.77	0.990
	Water 2	4.76	0.88	0.996	6.52	0.84	0.997	9.37	0.77	0.995	11.77	0.78	0.983
	Water 3	15.96	0.63	0.989	22.03	0.61	0.980	29.92	0.61	0.974	25.61	0.65	0.966

**Table 8:** Diffusional coefficient (D/cm^2^s^−1^).

Dose (kGy)	Water Type	0.5% NaAlg		1.0% NaAlg		1.5% NaAlg		2.0% NaAlg	
		D × 10^3^	R^2^	D × 10^3^	R^2^	D × 10^3^	R^2^	D × 10^3^	R^2^
12.5	Water 1	1.29	0.996	1.49	0.996	2.04	0.998	1.92	0.989
	Water 2	1.86	0.984	1.30	0.989	1.13	0.996	1.25	0.998
	Water 3	0.73	0.998	1.15	0.995	0.68	0.990	1.54	0.996
15	Water 1	0.86	0.995	1.29	0.997	1.65	0.995	1.74	0.992
	Water 2	1.52	0.986	0.72	0.993	1.04	0.997	1.07	0.995
	Water 3	0.31	0.995	0.61	0.996	0.60	0.993	1.08	0.997
17.5	Water 1	0.71	0.995	1.19	0.996	1.36	0.994	1.68	0.990
	Water 2	1.28	0.987	0.75	0.993	0.91	0.994	0.89	0.999
	Water 3	0.26	0.998	0.44	0.996	0.41	0.999	0.85	0.998
20	Water 1	0.48	0.998	0.67	0.997	1.14	0.995	1.60	0.993
	Water 2	0.73	0.993	0.64	0.993	0.89	0.998	0.65	0.997
	Water 3	0.19	0.997	0.25	0.995	0.26	0.999	0.52	0.976
25	Water 1	0.15	0.999	0.35	0.991	0.56	0.995	0.74	0.998
	Water 2	0.76	0.993	0.73	0.994	0.49	0.998	0.95	1.000
	Water 3	0.13	0.996	0.19	0.989	0.33	0.985	0.45	0.978

**Table 9:** NaAlg-g- AA hydrogels synthesis details.

Solutions Codes	Amount of Chemicals (g/100 mL Solution)		
	SA	AA	PP
Sol. 1	0.5	20	0.1
Sol. 2	1.0		
Sol. 3	1.5		
Sol. 4	2.0		

## Data Availability

Not applicable.
